# Innovative sphincterotomy knife and indigo carmine strategy for bile duct stones in a patient with surgically altered anatomy

**DOI:** 10.1055/a-2599-7007

**Published:** 2025-05-28

**Authors:** Koichi Soga, Kazuma Sakakibara, Yuki Soma, Fuki Hayakawa, Mayumi Yamaguchi, Ikuhiro Kobori, Masaya Tamano

**Affiliations:** 1 26263 Department of Gastroenterology, Dokkyo Medical University Saitama Medical Center, Koshigaya, Japan

Endoscopic treatment of common bile duct stones (CBDS) in patients with surgically altered anatomy is often difficult 1 . We describe a case of CBDS in a patient with surgically altered anatomy who we were able to safely treat by applying two innovative procedures.

An 85-year-old man had undergone distal gastrectomy and Roux-en-Y reconstruction for gastric cancer 4 years previously. He was referred to our hospital for endoscopic treatment of CBDS with controlled cholangitis after percutaneous transhepatic gallbladder drainage (PTGBD) was performed at another hospital ( Fig. 1 ).

**Fig. 1:**
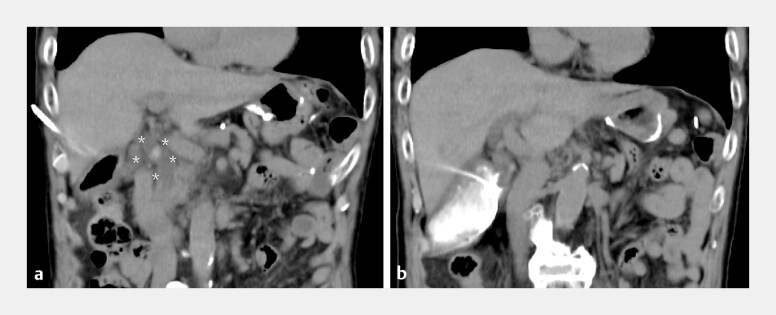
Abdominal computed tomography (CT) images. **a** An abdominal CT image obtained at another hospital showed common bile duct stones (asterisk). **b** A percutaneous transhepatic gallbladder drainage tube was placed to treat cholangitis caused by the common bile duct stones.

The CBDS were treated using a single-balloon enteroscope (SIF-H290S; Olympus, Tokyo, Japan). Papillary identification was simple, and we could intubate the pancreatic duct. However, we encountered difficulty because of the patient’s respiratory variability and the inability to fix the endoscope in a retroflex position 2 . After pancreatic duct cannulation, we performed two procedures that allowed complete CBDS extraction safely and rapidly.

First, we used a new endoscopic sphincterotomy (EST) knife (ENGETSU; Kaneka, Osaka, Japan) to create an incision for endoscopic pancreatic sphincterotomy. By rotating the EST knife to the left or right, it could easily be tensed or deflected, allowing the surgeon to make precise and appropriate incisions 3 . Additionally, this novel device allowed precise EST in three dimensions.

Second, we identified the bile duct opening using indigo carmine. Indigo carmine causes the color of bile to change, thereby allowing easy identification of the bile duct 4 . We mixed the indigo carmine with contrast medium and injected it into the bile duct through the PTGBD tube, which enabled identification of the bile duct orifice 5 ( Fig. 2 , Fig. 3 , Video 1 ).

**Fig. 2:**
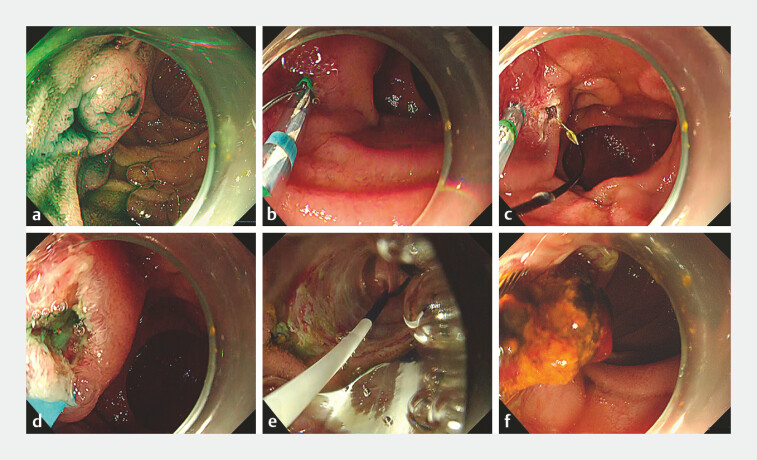
Retrieval of common bile duct stones (CBDS) using a single-balloon enteroscope (SIF-H290S; Olympus, Tokyo, Japan). **a** After pancreatic duct cannulation, we performed two procedures to safely and quickly complete CBDS removal. **b, c** First, a new endoscopic sphincterotomy (EST) knife (ENGETSU; Kaneka, Osaka, Japan) was used to create an incision for endoscopic pancreatic sphincterotomy. The EST knife can be rotated to the left or right by pushing or pulling, thus allowing the creation of an appropriate incision. **d** Second, the bile duct opening was identified using indigo carmine, which was mixed with contrast medium and injected into the percutaneous transhepatic gallbladder drainage tube. **e, f** The CBDS were removed endoscopically without complications.

**Fig. 3:**
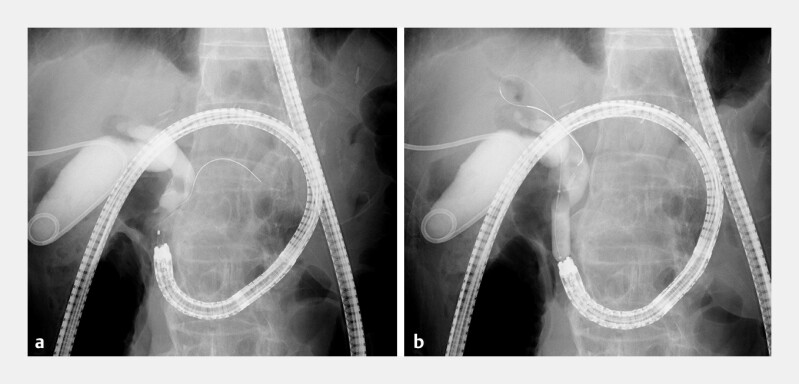
Identification of the bile duct opening using indigo carmine and fluoroscopy. **a** Cannulation of the bile duct was difficult; therefore, a guidewire was inserted into the pancreatic duct, and an endoscopic pancreatic sphincterotomy incision was created with a novel endoscopic sphincterotomy knife. **b** The indigo carmine was mixed with contrast medium and injected into the percutaneous transhepatic gallbladder drainage tube, which allowed easy identification of the bile duct stones and orifice. Endoscopic balloon dilation was performed after bile duct cannulation.

Innovative sphincterotomy knife and indigo carmine strategy for bile duct stones in a patient with surgically altered anatomy.Video 1

When performing endoscopic procedures on patients with surgically altered anatomy, we strive for both safety and precision, yet we often encounter significant challenges. By performing these two techniques, we were able to safely and rapidly treat the CBDS despite the surgically altered anatomy.

Endoscopy_UCTN_Code_TTT_1AR_2AC

## Notes

Endoscopy E-Videos https://eref.thieme.de/e-videos
*E-Videos* is an open access online section of the journal *Endoscopy* , reporting on interesting cases and new techniques in gastroenterological endoscopy. All papers include a high-quality video and are published with a Creative Commons CC-BY license. Endoscopy E-Videos qualify for HINARI discounts and waivers and eligibility is automatically checked during the submission process. We grant 100% waivers to articles whose corresponding authors are based in Group A countries and 50% waivers to those who are based in Group B countries as classified by Research4Life (see: https://www.research4life.org/access/eligibility/ ). This section has its own submission website at https://mc.manuscriptcentral.com/e-videos .
